# The Effect of Astragaloside on Pacemaker Current and the Cytoskeleton in Rabbit Sinoatrial Node Cells Under the Ischemia and Reperfusion Condition

**DOI:** 10.3389/fphar.2018.00551

**Published:** 2018-05-30

**Authors:** Ruxiu Liu, Jie Li, Yu Liu, Jie Peng, Xuanke Guan

**Affiliations:** Department of Cardiology, Guang’anmen Hospital, China Academy of Chinese Medical Sciences, Beijing, China

**Keywords:** sinoatrial node cells of neonatal rabbits, patch clamp, pacemaker current, astragaloside, ischemia/reperfusion

## Abstract

**Objective:** We investigated the role of astragaloside in the treatment of sick sinus syndrome (SSS).

**Methods:** Neonatal New Zealand rabbits were selected for the study. Rabbit sinoatrial node (SAN) cells were isolated by the method of dual enzymatic digestion and differential adherence. The injury model was prepared through simulated ischemia and reperfusion (I/R), and changes in the pacemaker current (*I*_f_) were recorded using the whole-cell patch-clamp technique. The proteins F-actin and vinculin were examined between various groups of SAN cells using a microplate reader and laser scanning confocal microscopy. The mRNA level and protein expression of hyperpolarization-activated cyclic nucleotide gated potassium channel 4 (HCN4) were assessed by q-PCR and western blot method.

**Results:** The peak current density of *I*_f_ was decreased to -19.64 ± 2.14 pA/pF in SAN cells after simulated I/R, and the difference was highly significant (*P* < 0.01). Following simulated I/R, 100, 200, or 300 μmol L^-1^ astragaloside was added to the extracellular solution of SAN cells; the peak current density of the *I*_f_ increased to -30.43 ± 1.98, -34.83 ± 1.6, and -52.72 ± 1.7 pA/pF, respectively (*P* < 0.01). Adding 100 μmol L^-1^ astragaloside to normal SAN cells also led to an enhanced peak current density of the *I*_f_ (*P* < 0.05). In a concentration-dependent manner, especially at 300 μmol/L, astragaloside was capable of increasing the expression of HCN4 and protecting the structural stability of F-actin and vinculin in the damaged SAN cells.

**Conclusion:** We estimated that astragaloside could shorten the action potential duration 20 (APD20) and APD50 in damaged SAN cells of neonatal rabbits, thereby increasing the expression of HCN4 and the *I*_f_ current density in damaged SAN cells of neonatal rabbits in a voltage-dependent manner, accelerating the steady-state activation of the *I*_f_ channels, and protecting damaged cytoskeleton.

## Introduction

Sick sinus syndrome (SSS) refers to a collection of disorders marked by the heart’s inability to perform its pacemaking function. It comprises various arrhythmias, including bradyarrhythmias with or without accompanying tachyarrhythmias. There is currently no elective drug therapy for SSS in western medicine. Treatment of sick sinus syndrome includes removing extrinsic factors, when possible, and pacemaker placement. Pacemakers do not reduce mortality, but they can decrease symptoms and improve the quality of life (Roginska and Bieganowska, 2014). In addition, pacemakers are expensive and the existence of multiple contradictions and complications has prevented their widespread application. In contrast, traditional Chinese medicine (TCM) is a reliable and cost-elective method for the treatment of SSS.

A large number of studies have shown that the HCN4 gene and its pacemaker current are closely related to sick sinus syndrome (Milanesi et al., 2006; Milano et al., 2014). The pacemaker current (*I*_f_) channel plays a very important role in the maintenance of sinoatrial node (SAN) automaticity. Studies have shown that the development of SSS is directly associated with structural damage as well as decreased SNC activity and automaticity, of which the molecular basis is the reduced automaticity caused by the structural abnormality of the *I*_f_ channel (Verkerk and Wilders, 2014). The activity of HCN4, the major component of the *I*_f_ channel, is dependent on its phosphorylation level, which is regulated by the protein kinase A (PKA) signaling pathway (Verkerk and Wilders, 2015).

Astragaloside is one of the most common traditional Chinese medicines and is derived from the herb *Astragalus membranaceus*. It has very extensive pharmacological activities, such as antibacterial, antipyretic, antipruritic, and antiarrhythmic activities. Its antiarrhythmic activity is one of its most remarkable activities (Wang et al., 2016). Previous pharmacologic studies on astragaloside showed that it significantly inhibits the widening of the QRS complex induced by TCM toad venum (Venenum Bufonis), indicating that astragaloside can alleviate Venenum Bufonis-induced ventricular arrhythmias in mice (Yu et al., 2006). Furthermore, astragaloside increased the activity of sodium-potassium adenosine triphosphatase (Na^+^-K^+^ ATPase) in the nerves and red blood cells of diabetic mice, reduced Ca^+^ concentrations in cardiac cells after ischemia and reperfusion (I/R) (Li and Cao, 2002), and inhibited K^+^ and Na^+^ channel currents in nerve cells (Zhu et al., 2008). The present study attempts to investigate the effect of astragaloside on the HCN4 gene and pacemaker current (*I*_f_) in SAN cells of neonatal rabbits and directly reveal the electrophysiological mechanisms of astragaloside in the treatment of bradyarrhythmia and sick sinus syndrome.

## Materials and Methods

### Experimental Animals

Male or female neonatal New Zealand big-eared white rabbits at <1 day of age were provided by the Experimental Animal Center of Guang An Men Hospital.

### Drugs and Solutions

Astragaloside was purchased from the Chinese National Institutes for Food and Drug Control (lot number: 110781-201314). Dulbecco’s modified Eagle’s medium (DMEM) was purchased from Invitrogen (United States); fetal bovine serum (FBS) and trypsin were purchased from GIBCO; collagenase type II, Triton X-100, and ethylenediaminetetraacetic acid disodium salt (EDTA-Na_2_) were purchased from Sigma-Aldrich; fluorescein isothiocyanate (FITC)-labeled phalloidin and monoclonal anti-vinculin antibody were purchased from Sigma-Aldrich (United States); and rhodamine tetramethylrhodamine isothiocyanate (TRITC)-conjugated goat anti-mouse immunoglobulin G (IgG) (H ± L) was purchased from BioWorld Products (United States).

A simulated ischemic solution (in mmol L^-1^) was prepared as follows: NaCl 98.5, KCl 10, NaH_2_PO_4_ 0.9, NaHCO_3_ 6.0, CaCl_2_ 1.8, MgSO_4_ 1.2, sodium lactate 40, and HEPES [4-(2-hydroxyethyl)piperazine-1-ethanesulfonic acid buffer; pH adjusted to 6.8 with 1% hydrochloric acid].

A simulated reperfusion solution was prepared as follows (in mmol L^-1^): NaCl 129.5, KCl 5.0, NaH_2_PO_4_ 0.9, NaHCO_3_ 20, CaCl_2_ 1.8, MgSO_4_ 1.2, glucose 55, HEPES 20 (pH adjusted to 7.4 with 1 mmol L^-1^ NaOH).

The perfusate for the pacemaker ionic current was prepared as follows (in mmol L^-1^): NaCl 137.0, KCl 5.4, CaCl_2_ 1.8, MgCl_2_ 0.5, NaHCO_3_ 23.8, NaH_2_PO_4_ 0.4, glucose 10 (pH adjusted to 7.4 with KOH).

The pipette solution for pacemaker ionic current was prepared as follows (in mmol L^-1^): NaCl 6.0, MgCl_2_ 1.0, EGTA 10, HEPES 5.0, KCl 140.0 (pH adjusted to 7.2 with KOH).

### Isolation, Purification, and Culture of SAN Cells From Neonatal Rabbits

A total of 75 neonatal New Zealand rabbits (<1 day) were selected. SAN cells were isolated from five rabbits for each procedure, and the procedure was repeated 15 times. The rabbits were disinfected with 75% ethanol, anesthetized by isoflurane, and then the heart was exposed. A dissecting microscope was used to collect tissue blocks (2 mm × 2 mm × 2 mm) from the venous sinus in the middle portion of the crista terminalis and root of the anterior vena cava, and then the tissue was placed in DMEM. Next, the tissue blocks were washed with phosphate-buffered saline (PBS) and cut into small pieces (0.3 mm × 0.3 mm × 0.3 mm). The supernatant was removed by aspiration, and the minced tissue pieces were digested with 8 mL of 0.08% trypsin for 5 min in a 37°C water bath under constant shaking. After pipetting and centrifugation, the supernatant was removed by aspiration, and the tissue pieces were further digested with 8 mL of 0.025% collagenase II for 10 min in a 37°C water bath, vigorously pipetted for 1 min, and precipitated. The supernatants were collected and transferred to 50-mL centrifuge tubes containing 20 mL of DMEM with 15% FBS. The precipitated tissue pieces were digested once again for the same length of time following the same procedure described above. The tissue pieces were then digested for the fourth time for 8 min following the same procedure described above.

After digestion, the tissue pieces were filtered through 400 mesh metal sieves. The filtrates were transferred to centrifuge tubes and centrifuged at 940 r min^-1^ for 7 min. After centrifugation, the supernatants were discarded, and cells were resuspended in culture medium at a density of 1 × 10^5^ cells L^-1^. The cell suspension was inoculated into six small Petri dishes. A trypan blue exclusion test showed that the cell viability exceeded 95%. The Petri dishes were incubated at 37°C in a humidified environment at 5% CO_2_ for 90 min. Fibroblasts were removed through differential adherence, and the remaining cells continued to grow in culture in the presence of 5-bromo-2′-deoxyuridine (5-BrdU; final concentration 0.1 mmol L^-1^). After 24 h of cultivation, the spent medium was replaced and then changed every other day afterwards, with 0.1 mmol L^-1^ 5-BrdU included in the fresh medium.

### Establishment of a Simulated I/R Model

The I/R model was constructed according to the method developed by Xiang et al. (2013). Ischemia was simulated by oxygen-glucose deprivation (OGD), whereas reperfusion was simulated through restoration of the oxygen and sugar supply. To simulate ischemia, culture medium was removed from the Petri dishes by aspiration, and the cells were washed three times using 2 mL of simulated ischemia solution that was pre-saturated in 95% N_2_ + 5% CO_2_. The cells were then covered with 2 mL of ischemic solution and placed into a homemade sealable box. Approximately 10 L (30 times the volume of the box) of gas mixture containing 95% N_2_ and 5% CO_2_ was passed through the box to fully expel the residual oxygen. Subsequently, the inlet and outlet tubing on the box were clamped shut with hemostat clamps. The cells (in the sealed box) were then placed in a 5% CO_2_ incubator and cultured under oxygen-glucose deprivation (OGD) conditions for 1 h to simulate ischemia. To simulate reperfusion, the homemade sealed box was removed from the incubator, and the ischemic solution in the Petri dishes was removed by aspiration. The cells were washed three times with 2 mL of simulated reperfusion solution containing 10% FBS. Subsequently, 2 mL of simulated reperfusion solution was added to the cells to restore the supply of oxygen and glucose, and the cells were cultured for 3 h in an incubator supplemented with 5% CO_2_. After the simulated reperfusion, SAN cells were subjected to patch-clamp experiments.

The cultured SAN cells were divided into five groups. The normal group included SAN cells incubated at 37°C. The culture medium was replaced with the simulated reperfusion solution, and a gas mixture containing 95% O_2_ and 5% CO_2_ was continuously passed into the solution. The simulated I/R group included cells cultured following the same procedure described above. The two groups of cells were pre-cultured in DMEM containing 15% blank serum for 30 min, and the cells were also divided into a high-dose astragaloside group, moderate-dose astragaloside group, and low-dose astragaloside group.

### Record of the Action Potential and Pacemaker Ionic Current in SAN Cells

All groups of cells were grown on microscope slides, transferred into a thermostatic perfusion chamber, and subject to continuous low-flow perfusion in the perfusion solution equilibrated with a gas mixture containing 95% O_2_ + 5% CO_2_. The electrode was manipulated to form a high impedance seal with the cells (>1.0 GΩ). Negative pressure suction was combined with pulse voltage to break the sealing diaphragms. The maximum diastolic potential of the SAN cells was recorded under the current-clamp mode, whereas *I*_f_ was recorded under the voltage-clamp mode, and the action potential was recorded under the current-clamp mode. The pulse release was controlled by computer through the software package Pclamp (Axon Instrument, Inc.). The digital signals were converted into electrical signals using a DigiData 1200 digital-to-analog converter. The electrical signals were amplified using the Axon-200B amplifier and transduced into cells through electrode wires and microelectrodes. The electrical signals produced by the cells were converted into digital signals using an analog-to-digital converter, and these signals were recorded using the PClamp program and stored on the hard drive of a computer. Microelectrode tip impedance was 4–6 MΩ, and the filtering frequency was 2 KHz. The experimental temperature was controlled at 35°C, and the perfusion rate was 2 mL min^-1^.

### Observation Using Laser Confocal Microscopy

The SAN cells were cultured on 14-mm coverslips placed in 24-well plates. The cells were allowed to adhere to the culture surface, and the exponentially growing cells were selected for assay. After the culture medium was removed by aspiration, the cells were washed twice with PBS and then fixed with 4% paraformaldehyde for 10 min. After removal of paraformaldehyde by aspiration, the cells were permeabilized with 0.02% Triton X-100 (15 min, 37°C, in an incubator). The cells were washed three times with PBS. F-actin was examined by incubating the wells containing cells with 100 μl of FITC-labeled phalloidin (5 μg mL^-1^) for 40 min at room temperature in the dark. The stained cells were mounted in buffered glycerol and examined under a microscope. Vinculin was examined by adding 100 μl of monoclonal anti-vinculin antibody (diluted 1:500 in PBS solution) to each well of cells, placing the cells into a humidified box, and incubating the cells at 4°C overnight. The cell were then washed twice with PBS and incubated with 100 μl of TRITC-conjugated goat anti-mouse IgG (H + L) (diluted 1:200 in PBS solution) for 1 h at room temperature. The stained cells were mounted in buffered glycerol and examined under a microscope. After the images were obtained, the mean fluorescence intensity was analyzed using the software Image-Pro Plus 6.0.

### The Effect of Expression of HCN4 mRNA Was Detected by RT-PCR

The TRIzol (Invitrogen, Carlsbad, CA, United States) method was used to extract the total RNA of cardiomyocytes in each group according to the kit instructions. In the catalysis of the reverse transcriptase, cDNA was synthesized by reverse transcription using the total RNA as a template in each group and oligo(dT) as a primer. According to RevertAid^TM^ First Strand cDNA Synthesis Kit (Fermentas) instructions, 5.0 μg total RNA was reserved for cDNA with oligo(dT) 18 as a primer, and the reaction volume was 20 μL. In accordance with the SYBR Green PCR Master Mix (Applied Biosystems) instructions, 2.0 μL cDNA was reserved for RT-PCR detection, and PCR was required for the enzyme and buffer system for the SYBR Green PCR Master Mix kit, containing 12.5 μl 2x SYBR Green PCR Master Mix in a total reaction volume of 25 μL. A total volume of 2 μL was used for the upstream and downstream primers, 2 μL of cDNA was used, and 8.5 μl of nuclease-free H_2_O was used. According to the gene nucleic acid sequence provided by the American Biological Gene Library, the 50% G + C content with no hairpin structure was selected as the primer sequence, and there were no homologous sequences of two conserved sequences opposite each other, as designed by Primer Premier 5.0 software, and synthesized by Beijing Parkson Corporation. The primer sequences were as follows: (reference gene 263 bp), upstream: 5-GAG ACC TTC AAC ACC CCA GCC-3; downstream: 5-AAT GTC ACG CAC GAT TTC CC-3; HCN4 target gene 74 bp, upstream: 5-GCTGTCCTGTCGTCTTGTTTCTC-3, downstream: 5′-CGGTTATTGTTGGTGGTGTATCTC-3′. Reverse transcription: 42°C water bath for 60 min, and 70°C water bath for 10 min. Next, PCR was conducted with the GeneAmp 5700 fluorescence quantitative PCR instrument using the following cycle parameters: 95.0°C 10 min (one cycle); 95.0°C, 25 s, 55.0°C, 25 s, 72.0°C, 50 s, one cycle; 72.0°C 5 min (one cycle). A computer was connected to the PCR instrument and implemented the reactions according to the above parameters, and each cycle was automatically recorded by the computer in accordance with the fluorescence signal values in the reaction tube, and curved. At the end of the reaction, the results were analyzed, and the fixed values were automatically quantified by machine software. The results are represented as the threshold (threshold cycle, *C*t), which is the number of circulations of fluorescence signal to setting the threshold value in each reaction tube; Δ*C*t = *C*t_HCN4_ -*C*t_beta-actin_; before and after treatment, the relative change of the same gene was adopted by the 2^-ΔΔ^*^C^*^T^ method (Roginska and Bieganowska, 2014).

### The Effect of Expression of HCN4 Was Detected by Western Blot

Western blot was used to investigate changes of HCN4. Total Protein was obtained from the SNC. Protein samples were separated on 8 and 12% SDS-PAGE gels and transferred onto nitrocellulose membranes. The protein on the gel was transferred to a PVDF membrane, mounted with 5% skim milk powder at room temperature for 1 h, and incubated with primary antibody at 4°C overnight. Chemioluminescent detection was performed with substrate reagents (Solarbio, Germany). The data were analyzed by densitometric analysis with Image J software.

### Statistical Analysis

The obtained data were processed using Origin statistical software, and the measured data are expressed as 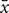 ±*s*. Multigroup comparisons were performed using one-way analysis of variance (ANOVA), univariate continuous data were analyzed using a *t*-test, and count data were analyzed using a *X*^2^ test. *P*-values less than 0.05 were considered statistically significant.

## Results

### The Morphology of SAN Cells

**FIGURE 1:**
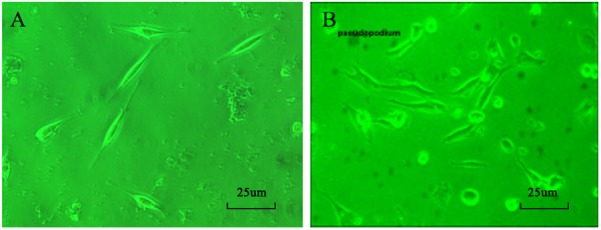
**(A)** Morphology of normal SAN cells (200×). **(B)** Morphology of SAN cells after establishment of the model (200×).

Cell morphology was examined under an inverted microscope, and SAN cells appeared spindle-shaped. The nuclei of the SAN cells were medium-sized, oval, and located in the center of the cells. The cell membrane was smooth, intact, and in excellent condition (**Figure 1A**). After simulating I/R, the majority of the SAN cells underwent apoptosis. The surviving SAN cells remained attached to the culture surface; however, cell swelling, cell deformation, pseudopod retraction, and cell detachment were clearly visible. In addition, a large number of particles was found on the cell surface, and the surface appeared rough (**Figure 1B**).

### Effect of Astragaloside on the APD20, APD50, APD90 of Action Potential in the Damaged SAN Cells of Neonatal Rabbits

The APD20 was 25.7 ± 4.8 ms in normal SAN cells. After the simulated I/R, the APD20 was extended to 82.6 ± 5.3 ms. The difference was statistically significant (*P* < 0.05).

**FIGURE 2:**
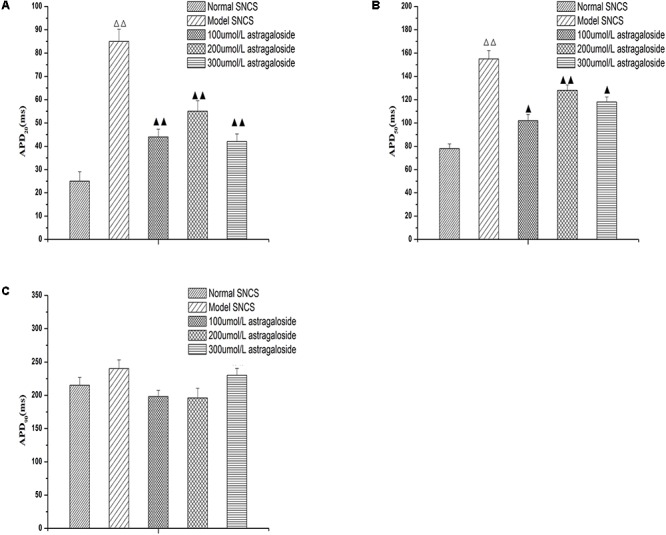
**(A)** Effect of astragaloside on the APD20 in the damaged SAN cells of neonatal rabbits. **(B)** Effect of astragaloside on the APD50 in the damaged SAN cells of neonatal rabbits. **(C)** Effect of astragaloside on the APD90 in the damaged SAN cells of neonatal rabbits. *n* = 10, ^
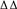
^*P* < 0.01 vs. SAN cells; ^
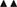
^*P* < 0.01, ^
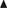
^*P* < 0.05 vs. SAN cells after establishment of the mode.

After simulating I/R, the SAN cells were treated with 100, 200, and 300 μmol L^-1^ astragaloside, and the treatments resulted in varied shortening of APD20 to 41.5 ± 5.6, 54.5 ± 5.4, and 41.3 ± 5.3 ms, respectively (**Figure 2A**). The difference was statistically significant (*P* < 0.05), but there was no statistical difference between the three doses.

The APD50 was 78.79 ± 5.3 ms in normal SAN cells. After the simulated I/R, the APD20 was extended to 152.5 ± 5.6 ms (*P* < 0.01). After simulating I/R, the SAN cells were treated with 100, 200, and 300 μmol L^-1^ astragaloside, and the treatments resulted in varied shortening of APD50 to 97.8 ± 5.6, 124.6 ± 4.6, and 118.5 ± 3.6 ms, respectively (*P* < 0.01) (**Figure 2B**). The 100 μmol/L group was clearly shorter than the 200 and 300 μmol/L group (*P* < 0.05), but there was no statistically significant difference between the 200 and 300 μmol/L group. Compared with the normal group, the APD90 was extended after the simulated I/R without statistical significance (*P* > 0.05). After simulating I/R, the SAN cells were treated with 100, 200, and 300 μmol L^-1^ astragaloside, but there was no statistically significant difference (**Figure 2C**) (*P* > 0.05). These results suggested that astragalus shortened the APD20, APD50, and APD90, and improved the spontaneous beat frequency in the damaged SAN cells. There was no significant effect on APD90.

### Effect of Astragaloside on the Peak Current of the *I*_f_ in the Damaged SAN Cells of Neonatal Rabbits

#### Effect of Astragaloside on the Current–Voltage (*I–V*) Curves of the *I*_f_ in Neonatal Rabbits

**FIGURE 3:**
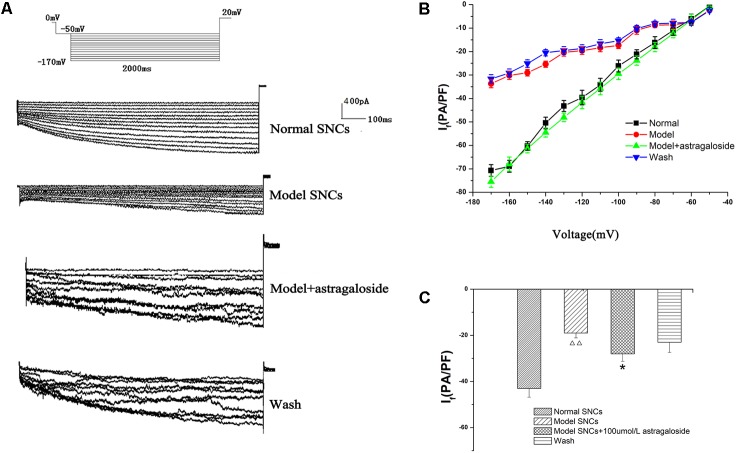
**(A)** Representative *I*_f_ traces recorded from the different groups. **(B)** The current–voltage (*I–V*) curves of each group. **(C)** The peak current densities in each group exhibited significant differences. *n* = 10, ^
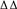
^*P* < 0.01 vs. the normal group. ^∗^*P* < 0.05 vs. the I/R model group.

The *I*_f_ values corresponding to various voltages (-50 ∼-170 mV) were measured in each group of SAN cells, and the current densities were calculated and *I–V* curves were constructed. It can be seen from the *I*_f_ traces of each group that the amplitude of *I*_f_ in the I/R model group was significantly decreased compared with the normal group (**Figure 3A**). After treatment with 100 μmol L^-1^ astragaloside in the I/R model group, *I*_f_ was significantly increased (**Figure 3A**). After washing, compared with the astragaloside group, the amplitude of *I*_f_ in the washed group was significantly reduced.

The *I–V* curve is also shown, and compared with the normal group, under voltages below -70 mV, the decrease in current density in the I/R model group was statistically significant (*P* < 0.05). However, the shape of the *I–V* curves remained essentially the same. Astragaloside treatment resulted in varying degrees of increased current density under various voltages. Under a voltage range of -70 ∼-170 mV, the recovery of the *I–V* curve was particularly evident. Compared with that of the model group, the difference was statistically significant (*P* < 0.05) (**Figure 3B**). The results showed that after establishing the simulated I/R model, the current density of the *I*_f_ was decreased in the SAN cells under various voltages. The decrease was even more pronounced upon hyperpolarization (below -70 mV). Astragaloside increased the current density of the *I*_f_ in the SAN cells after establishing the model. The increase was especially significant upon hyperpolarization (below -80 mV), and the results indicated that the I/R and astragaloside-induced changes in the current density of the *I*_f_ were voltage dependent.

The peak current density of the *I*_f_ in normal SAN cells and the I/R model group was -43.48 ± 1.08 pA/pF and -19.64 ± 2.14 pA/pF, respectively (*P* < 0.01). After treatment with astragaloside in the I/R model group, the peak current density of the *I*_f_ was raised to -30.43 ± 1.98 pA/pF (*P* < 0.01). After washing, compared with the astragaloside group, the peak current density of the *I*_f_ was reduced to -23.44 ± 1.87 pA/pF (*P* < 0.01, **Figure 3C**). The above results demonstrate that the simulated I/R reduced the peak current density of the *I*_f_ in the SAN cells of neonatal rabbits. Astragaloside increased the peak current density of the *I*_f_ in normal and damaged SAN cells of neonatal rabbits and reversed the I/R-induced *I*_f_ changes in SAN cells to a certain extent.

#### Effect of Astragaloside on the Steady-State Activation (SSA) Curves of the *I*_f_ in Neonatal Rabbits

**FIGURE 4:**
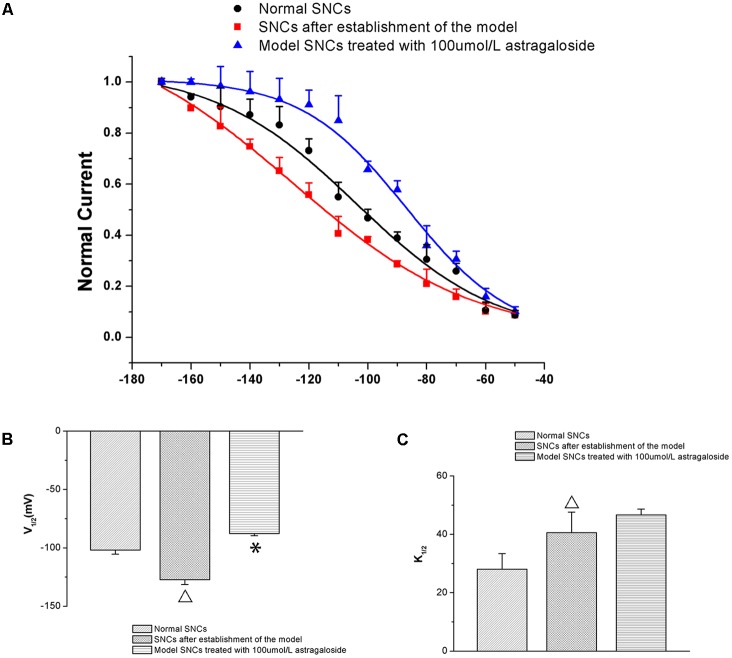
**(A)** Differences in the SSA curves of the *I*_f_ in each group (*n* = 10). **(B)** Effect of astragaloside on the *V*_1/2_ of the *I*_f_ in damaged neonatal rabbit SAN cells. ^∗^*P* < 0.05 vs. the normal group; ^
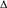
^*P* < 0.05 vs. the I/R model group. **(C)** Effect of astragaloside on the slope factor k of the half activation voltage of the *I*_f_ in damaged SAN cells. ^∗^*P* < 0.05 vs. the normal group; ^
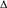
^*P* < 0.05 vs. the I/R model group.

Compared with that of the normal group, the SSA curve of the *I*_f_ was left-shifted toward more negative potentials in SAN cells after establishing the simulated I/R model. The half-maximal activation voltage (*V*_1/2_) was -101.87 ± 3.48 mV in the normal group and -127.25 ± 4.08 mV in the model group (*P* < 0.05). After adding 100 μmol L^-1^ astragaloside, the SSA curve shifted to the right and the *V*_1/2_ -87.74 ± 1.91 mV. Compared with that of the model group, the difference was statistically significant (*P* < 0.05). After adding 100 μmol L^-1^ astragaloside, the *k*-value was changed to 46.62 ± 2.01 mV. Compared with that of the model group, the difference was statistically significant (*P* < 0.05). The results indicated that the simulated I/R decelerated the SSA of the *I*_f_ channels in the SAN cells of neonatal rabbits. Under the same depolarizing voltage, channel activation was delayed, resulting in reduced current densities in the channels. In contrast, the astragaloside treatment accelerated the activation of the *I*_f_ channels and restored the current density, thereby shortening the diastolic depolarization phase of the action potential in SAN cells and improving the autorhythmicity of SAN cells (**Figure 4**).

#### Time-Dependence of the Effects of Astragaloside on the *I*_f_ in Neonatal Rabbits

**FIGURE 5:**
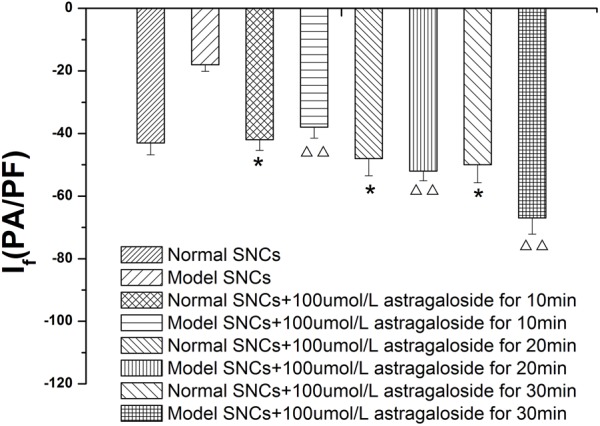
Time dependence of the effects of astragaloside on the peak current density of the *I*_f_ in normal SAN cells. *n* = 10, ^∗^*P* < 0.05 vs. the normal group. ^
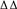
^*P* < 0.01 vs. SAN cells after establishment of the model.

The *I*_f_ values were measured in normal SAN cells under the maximum voltage (-170 mV) at 10, 20, and 30 min after the addition of 100 μmol L^-1^ astragaloside, and the experimental results showed that the *I*_f_ values were -42.45 ± 3.25 pA/pF (*P* > 0.05), -44.68 ± 2.57 pA/pF (*P* > 0.05), and -49.28 ± 3.15 pA/pF (*P* < 0.05), respectively). The *I*_f_ values were also measured in SAN cells after simulating I/R and treatment with astragaloside. Under the maximum voltage (-170 mV), the *I*_f_ values at 10, 20, and 30 min after the addition of 100 μmol L^-1^ astragaloside were -38.05 ± 2.56, -52.89 ± 2.59, and -67.87 ± 2.18 pA/pF, respectively (*P* < 0.01). Therefore, it can be concluded that astragaloside exerted a long-lasting activational effect on the *I*_f_ channels encoded by the HCN4 gene. (**Figure 5**).

#### Concentration Dependence of the Effects of Astragaloside on the *I*_f_ in Neonatal Rabbits

**FIGURE 6:**
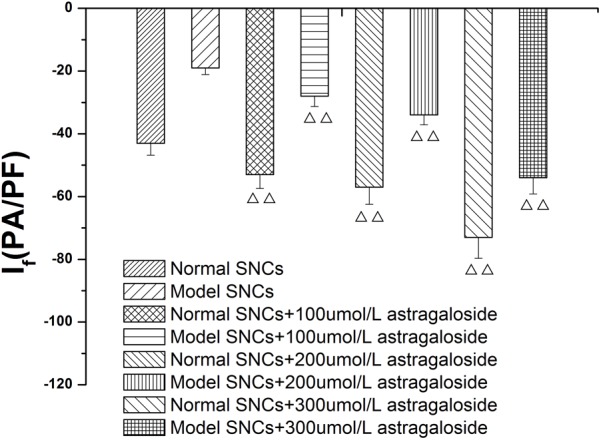
Concentration dependence of the effects of astragaloside on the peak current density of the *I_f_* in normal SAN cells and damaged SAN cells. *n* = 10, ^
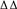
^*P* < 0.01, vs. SAN cells after establishment of the model.

In the present study, different concentrations of astragaloside (100, 200, and 300 μmol L^-1^) were sequentially added to normal SAN cells. At 5 min after addition of astragaloside, the peak current densities of the *I*_f_ were measured under the maximum voltage (-170 mV). The results showed that the peak current densities of the *I*_f_ were -53.41 ± 1.42, -59.59 ± 2.29, and -74.26 ± 2.07 pA/pF, respectively (*P* < 0.01). In addition, 100, 200, and 300 μmol L^-1^ astragaloside were added to SAN cells after the simulated I/R, and the peak current densities of the *I*_f_ were determined. Astragaloside treatments increased the peak current densities of the *I*_f_ to varying extents, and the current densities reached -30.43 ± 1.98, -34.83 ± 1.6, and -52.72 ± 1.7 pA/pF (*P* < 0.01), respectively (**Figure 6**).

### Results of Laser Scanning Confocal Microscopy

**FIGURE 7:**
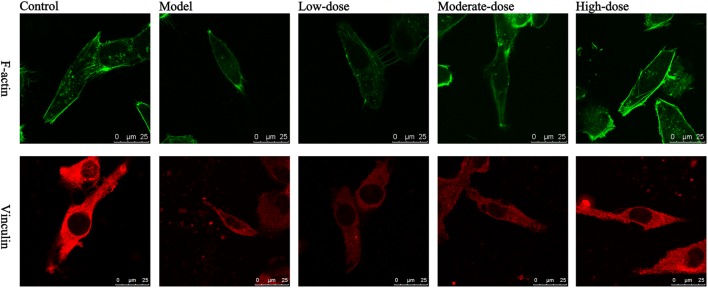
Laser confocal images of F-actin and vinculin in various groups of SAN cells. Green: F-actin; red: vinculin; scale bar: 25 μm.

The results of laser scanning confocal microscopy showed that the blank control group displayed apparent cytoskeletal staining. The cytoskeleton was intact, appeared evenly distributed, exhibited a clearly defined structure, and filled the entire cytoplasm. After simulating I/R, the cytoskeleton was arranged in a disordered manner in the SAN cells and the amount of cytoskeletal components was decreased. In addition, the mean fluorescence intensity was significantly reduced compared with that of the blank control group. Compared with the model group, the high-dose astragaloside-treated group exhibited an increased amount of cytoskeleton. In addition, the cytoskeleton in the high-dose group was arranged in a more orderly manner and the mean fluorescence intensity was significantly elevated (**Figure 7**). In the moderate-dose group, the cytoskeleton remained intact, although the amount of cytoskeleton was reduced compared with the blank control group, and the mean fluorescence intensity was significantly increased compared with the model group. Significant differences were not detected in the cytoskeletal morphology and mean fluorescence intensity between the low-dose group and the model group. The results indicated that astragaloside was capable of protecting the morphology and structure of the cytoskeletal proteins F-actin and vinculin in SAN cells after I/R. Moreover, a dose-effect relationship was detected (**Table 1**).

**Table 1:** Mean fluorescence intensity of the integrated optical density per stained area (IOD area^-1^) in various groups of SAN cells (*n* = 4).

Group	F-actin	Vinculin
Control group	7.835 ± 0.798	8.310 ± 0.362
Model group	4.740 ± 0.203^Δ^	4.780 ± 0.233^Δ^
High-dose group	7.105 ± 0.375^∗^	7.542 ± 0.419^∗^
Moderate-dose group	6.210 ± 0.216^∗^	6.180 ± 0.168^∗^
Low-dose group	4.902 ± 0.213	4.653 ± 0.338

*Compared with the control group, ^Δ^p < 0.01; compared with the model group, ^∗^p < 0.01.*

### The Effect of Astragaloside on the Expression of HCN4 mRNA

Compared with that of the normal group, the expression of HCN4 mRNA was significantly decreased in the model group, and the difference was statistically significant (*P* < 0.05 or *P* < 0.01). When comparing the astragaloside group with the model group, the expression of HCN4 mRNA of the high dose group was significantly increased, and it was clearly greater than that of the medium or low dose group, with a difference that is statistically significant (*P* < 0.05) (**Table 2**).

**Table 2:** mRNA expression of HCN4 in each group (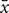 ± *s*).

Group	Number	2^-ΔΔ^	*^C^*^T^ (HCN4)
Control group	5	0.38 ± 0.44
Model group	5	0.16 ± 0.17^Δ^
High-dose group	5	0.79 ± 0.53^∗^
Moderate-dose group	5	0.36 ± 0.22^∗^
Low-dose group	5	0.17 ± 0.35

*Compared with the control group, ^Δ^p < 0.01; compared with the model group, ^∗^p < 0.01.*

### The Effect of Astragaloside on the Expression of HCN4

**FIGURE 8:**
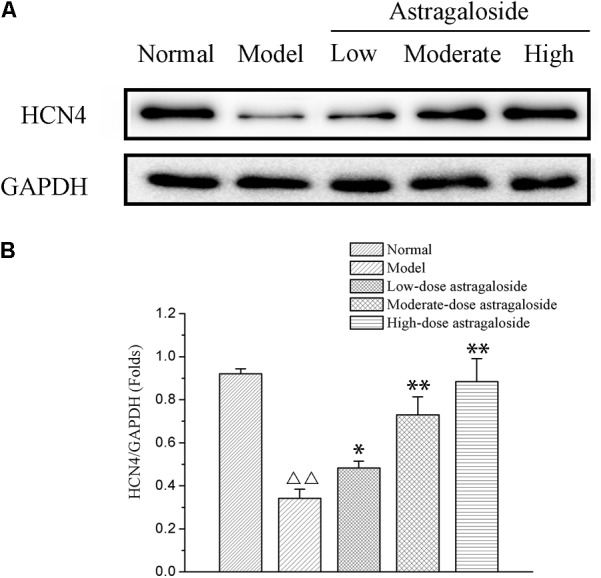
**(A)** The effect of astragaloside on the expression of HCN4. **(B)** Quantification of different group on the expression of HCN4 protein. Compared with the Normal group, ^ΔΔ^*p* < 0.01; compared with the model group, ^∗∗^*p* < 0.01, ^∗^*p* < 0.05.

Compared with that of the normal group, the expression of HCN4 was significantly decreased in the model group (*P* < 0.01). Compared with that of the model group, the expression of HCN4 of the high dose group was significantly increased, and it was clearly greater than that of the medium or low dose group, with a difference that is statistically significant (*P* < 0.05 or *P* < 0.01) (**Figure 8**).

## Discussion

The main findings of the current study are that (1) astragaloside shortened the APD20 and APD50 in damaged SAN cells of neonatal rabbits, and (2) astragaloside increased the *I*_f_ current density in a voltage-dependent manner and accelerated the steady-state activation of the *I*_f_ channels in damaged SAN cells of neonatal rabbits. (3) Additionally, astragaloside increased the expression of HCN4 and protected the damaged cytoskeleton in damaged SAN cells of neonatal rabbits.

Astragaloside is similar to the molecule cycloastragenol in chemical structure and exerts a variety of pharmacological effects. Studies have demonstrated its effect on cardiovascular systems, and it also enhances immune function with its anti-inflammatory, anti-viral, and anti-oxidation action, reduces hypoxic-ischemic myocardial injury, enhances myocardial contractility and diastolic action of coronary blood vessels, and expands coronary arteries (Murata et al., 2017). Hyperpolarization-activated cyclic nucleotide-gated channels in sinoatrial node cells play a decisive role in maintaining SAN cells via self-discipline. SAN cell structural damage, and active and self-discipline are directly related to the generation of SSS (Morad and Zhang, 2017). The main molecular basis of SSS is that *I*_f_ channel structural abnormalities lead to lower self-discipline due to the pacemaker current in the sinus node, which controls the rate and rhythm of the heart.

Studies to determine the effects of astragaloside on cardiomyocytes have primarily focused on calcium channels rather than on *I*_f_ (Zhao et al., 2015). The results of the present study show that astragaloside increased the *I*_f_ current density in the normal and damaged SAN cells of neonatal rabbits in a voltage-dependent manner. The SSA curve of the *I*_f_ channel was shifted to the right, indicating that the astragaloside treatment accelerated channel activation and restored the reduced current density. As a result, the diastolic depolarization phase of the action potential was shortened in SAN cells, and the autorhythmicity of SAN cells was improved. *I*_f_ plays an important role in regulating spontaneous cardiac rhythms and heart rate.

The destruction of the myocardial cytoskeletal system is the main mechanism underlying the occurrence of I/R injury. Studies have shown that microfilaments and microtubules become arranged in a disorderly manner in the human heart after 10 min of ischemia (Livak and Schmittgen, 2001). F-actin and vinculin are major components of the cytoskeleton, and cytoskeletal microfilaments that are mainly composed of F-actin lie beneath the cell membrane. The microfilaments exert a supportive effect on the cell membrane and participate in the maintenance of stable cell volume. Under hypotonic environments, water enters the cell and causes cell volume expansion. Cell volume is then gradually restored to a normal range through autonomous regulation.

Undrovinas et al. (1995) found that the treatment of rat and rabbit ventricular myocytes with cytochalasin D decreases Na^+^ current amplitudes. The results of single-channel assays show that the open probability of *I*_Na_ is reduced, and ankyrin B, which connects cytoskeletal microfilaments with *I*_Na_, has been reported as knocked out (Chauhan et al., 2000). The effect of ankyrin B knockout is reportedly similar to the effect of destroying cytoskeletal microfilaments, which indicates that the regulatory effect of cytoskeletal microfilaments on myocardial *I*_Na_ occurs at the molecular level. Wang et al. (2004) reported that the expression of Kv4.2 is increased after actin depolymerization, indicating that the microfilaments not only regulate the function of channels but also affect the expression of channels. Vinculin is associated with the actin cytoskeleton in cells and anchors the microfilaments to the cell membrane. Vinculin also mediates the connection between the extracellular matrix and cytoskeleton. Therefore, vinculin plays an important role in maintaining cell morphology and regulating cell adhesion, cell movement, cell proliferation, and signal transduction across the cell membrane (Ziegler et al., 2008).

The cell membrane, cytoskeleton, and interactions between the cell membrane and cytoskeleton are key factors in cell self-regulation and adaptation (Wei, 2005). Channel proteins in the cell membrane are connected to the cytoskeleton through adaptor proteins. The connection between the channel protein and cytoskeleton provides a structural basis for the interactions between adjacent channel proteins. The cell membrane “protects” the cytoskeleton by stabilizing the intracellular ion balance. In addition, the cell membrane regulates the rearrangement of the damaged cytoskeleton through stretch-activated ion channels and mechanosensitive enzymes. However, the cytoskeleton “protects” the cell membrane by providing structural support, promoting the stability of the stress-bearing area, and regulating the mechanosensitive ion channels. In addition, the cytoskeleton may be involved in the repair of the cell membrane after force-induced ruptures.

The experimental results show that astragaloside protects the structural stability of the cytoskeletal proteins F-actin and vinculin in damaged SAN cells, thereby maintaining the cell morphology. Astragaloside also acts to maintain the morphology, structure, and electrophysiological functions of SAN cells by regulating cell volume and *I*_f_. Moreover, a relationship exists between astragaloside dose and effect, and astragaloside may exert a therapeutic effect on sick sinus syndrome by protecting the cell structure and enhancing the *I*_f_.

## Ethics Statement

This study was carried out in accordance with the recommendations of institutional guidelines for the care and use of laboratory animals, Guang’anmen Hospital, China Academy of Chinese Medical Sciences. The protocol was approved by the Guang’anmen Hospital, China Academy of Chinese Medical Sciences.

## Author Contributions

RL, JL, YL, and JP contributed equally to this work. RL contributed to total experimental design. YL contributed to experimental implementation and article writing. JP contributed to experimental implementation. XG and JL contributed to article writing. All authors discussed the results and commented on the manuscript.

## Conflict of Interest Statement

The authors declare that the research was conducted in the absence of any commercial or financial relationships that could be construed as a potential conflict of interest.
